# Central obesity, smoking habit, and hypertension are associated with lower antibody titres in response to COVID‐19 mRNA vaccine

**DOI:** 10.1002/dmrr.3465

**Published:** 2021-05-11

**Authors:** Mikiko Watanabe, Angela Balena, Dario Tuccinardi, Rossella Tozzi, Renata Risi, Davide Masi, Alessandra Caputi, Rebecca Rossetti, Maria Elena Spoltore, Valeria Filippi, Elena Gangitano, Silvia Manfrini, Stefania Mariani, Carla Lubrano, Andrea Lenzi, Claudio Mastroianni, Lucio Gnessi

**Affiliations:** ^1^ Department of Experimental Medicine Section of Medical Pathophysiology, Food Science and Endocrinology Sapienza University of Rome Rome Italy; ^2^ Department of Endocrinology and Diabetes University Campus Bio‐Medico of Rome Rome Italy; ^3^ Department of Molecular Medicine Sapienza University of Rome Rome Italy; ^4^ Department of Public Health and Infectious Diseases Sapienza University of Rome Rome Italy

**Keywords:** BMI, immunogenicity, infection, SARS CoV‐2, vaccination, waist circumference

## Abstract

**Aims:**

To explore variables associated with the serological response following COVID‐19 mRNA vaccine.

**Methods:**

Eighty‐six healthcare workers adhering to the vaccination campaign against COVID‐19 were enrolled in January–February 2021. All subjects underwent two COVID‐19 mRNA vaccine inoculations (Pfizer/BioNTech) separated by 3 weeks. Blood samples were collected before the 1st and 1–4 weeks after the second inoculation. Clinical history, demographics, and vaccine side effects were recorded. Baseline anthropometric parameters were measured, and body composition was performed through dual‐energy‐X‐ray absorptiometry.

**Results:**

Higher waist circumference was associated with lower antibody (Ab) titres (*R* = −0.324, *p* = 0.004); smokers had lower levels compared to non‐smokers [1099 (1350) vs. 1921 (1375), *p* = 0.007], as well as hypertensive versus normotensive [650 ± 1192 vs. 1911 (1364), *p* = 0.001] and dyslipideamic compared to those with normal serum lipids [534 (972) vs 1872 (1406), *p* = 0.005]. Multivariate analysis showed that higher waist circumference, smoking, hypertension, and longer time elapsed since second vaccine inoculation were associated with lower Ab titres, independent of BMI, age. and gender.

**Conclusions:**

Central obesity, hypertension, and smoking are associated with lower Ab titres following COVID‐19 vaccination. Although it is currently impossible to determine whether lower SARS‐CoV‐2 Abs lead to higher likelihood of developing COVID‐19, it is well‐established that neutralizing antibodies correlate with protection against several viruses including SARS‐CoV‐2. Our findings, therefore, call for a vigilant approach, as subjects with central obesity, hypertension, and smoking could benefit from earlier vaccine boosters or different vaccine schedules.

## 1 INTRODUCTION

Since severe acute respiratory syndrome coronavirus 2 (SARS‐CoV‐2) was described in 2019, the world has faced an unprecedented pandemic causing millions of deaths. Obesity and excess visceral fat were shown to be major risk factors for the development of complications following COVID‐19 infection,^1^, ^2^, ^3^, ^4^ and, even before vaccines were made available, concerns regarding the possibility that obesity may blunt their efficacy were raised.^5^ In December 2021, Polack et al.^6^ reported promising results regarding an mRNA vaccine against COVID‐19, BNT162b2 (Pfizer, Inc., and BioNTech), which conferred 95% protection against COVID‐19 in adult subjects. The trial enrolled approximately 44,000 subjects, and there were 8 and 162 COVID‐19 cases, respectively, following the two doses of vaccine or placebo. Despite the population comprising 35% of patients with a BMI of more than 30, and the good efficacy data reported towards COVID‐19 infection, evidence regarding the ability to protect against severe COVID‐19 is less certain for subpopulations, and long‐term data are lacking. Moreover, obesity is not defined by BMI, but by the presence of fat excess,^7^ BMI often mistakenly including subjects with fat excess in the normal weight category and vice versa. Body fat measured through bioimpedentiometry (BIA) or dual X‐ray absorptiometry (DXA) may represent an alternative, but cut‐off values have never been validated, and the necessary equipment may not be promptly available in many clinical practices. The use of waist circumference as a measure of central obesity is, conversely, well established, and the European Association for the Study of Obesity, recommends to screen for obesity complications not only those with a BMI ≥ 25, but also those with central obesity (waist circumference ≥80 cm for women and ≥94 cm for men). To the best of our knowledge, no studies are available to date investigating the real‐life immunogenicity following BNT162b2 vaccine in relation to body composition and body fat distribution. In Italy, the BNT162b2 Pfizer BioNtech vaccine has been selected to be administered to healthcare professionals since December 2020. Our primary aim focused on exploring the association between the response to the COVID‐19 vaccine and adiposity parameters such as central obesity; as a secondary aim, we explored its possible association with other cardiometabolic conditions and risk factors.

## 2 MATERIALS AND METHODS

### 2.1 Population and study design

Subjects included in this single‐centre observational study were enrolled among healthcare workers of Policlinico Umberto I of Rome voluntarily undergoing a Pfizer BioNtech COVID‐19 vaccine in January and February 2021. All subjects had undergone repeat naso‐ and oro‐pharyngeal swabs as per hospital policy throughout the pandemic, and no previous infection had been recorded. Before enrolment, all subjects underwent a venous blood draw in order to confirm absence of antibodies against SARS‐CoV‐2. Those who had a positive serology were excluded from the study. The inclusion criteria were as follows: age over 18 years old, stable body weight (less than 5 kg self‐reported change during the preceding 3 months); absence of previous SARS‐CoV‐2 infection, absence of contraindications to the vaccine, willingness to undergo voluntary vaccination, absence of immunodepression, no use of medications known to impact the immune system, and no ongoing pregnancy. Data about demographic characteristics were collected with the means of a structured interview. The study was approved by the local IRB (prot. CE 6228), conducted in accordance with the Declaration of Helsinki and the Good Clinical Practice. Written informed consent was obtained from all study participants before enrolment.

### 2.2 Vaccination procedure and blood collection

All patients were subjected to two COVID‐19 vaccine inoculations, separated by 21 days (Comirnaty, Pfizer‐BioNTech). Before the first inoculation, all patients underwent a blood draw that was handled according to local standards of practice. A second blood draw was collected between 1 and 4 weeks after the second inoculation, 28–49 days after the first inoculation. Samples were centrifuged and plasma kept at −80°C until further analysis.

### 2.3 Biochemical measures

Routine biochemical tests were handled according to standard operating procedures. Anti‐SARS‐Cov‐2 antibodies were measured through a commercially available assay (Elecsys® Anti‐SARS‐CoV‐2 assay; Roche Diagnostics), which detects total antibodies against the SARS‐CoV‐2 spike (S) antigen in a sandwich electrochemiluminescence assay.^8^

### 2.4 Anthropometric and body composition assessment

Anthropometric parameters were measured at baseline. Body weight was measured using a balance‐beam scale (Seca GmbH & Co). Height was rounded to the closest 0.5 cm. BMI was calculated as weight in kilograms divided by squared height in metres (kg/m^2^). Waist circumference was measured midway between the lower rib and the iliac crest, hip circumference at the level of the widest circumference over the great trochanters to the closest 1.0 cm. The measurements were performed with the means of an anelastic tape by trained professionals. Body composition was measured through DXA (Hologic 4500) as previously reported.^9^

### 2.5 Statistics

The Statistical Package for Social Sciences (SPSS), v.20 was used for statistical analysis. Results are presented as mean, standard deviation (SD), or median, interquartile range (IQR) according to their distribution. Normality was assessed with the Kolmogorov–Smirnov test. Variables not normally distributed were log‐transformed. A Kruskal–Wallis test with a Bonferroni post‐hoc multiple comparison was conducted to compare the distribution of the COVID‐19 Ab titres among the four different levels (quartiles) of waist circumference. A Mann–Whitney *U* test was conducted to compare the distribution of the COVID‐19 Abs in subpopulations. Univariate and multivariate linear regression models were performed to analyse the relationship between the SARS‐CoV‐2 Ab as the dependent variable and clinical, biochemical, DEXA‐derived body composition parameters as the independent variables.

To build a multivariate linear regression model with Ab titres as the dependent variable, we used an enter method approach (all the independent variables included in the same regression equation) and investigated the following variables/models: (1) multivariate analysis including age and BMI together with variables with significant univariate association (*p* ≤ .05) analysed one by one as regressors (age + BMI + Waist circumference; age + BMI + waist to hip ratio; age + BMI + time; age + BMI + hypertension; age + BMI + dyslipidaemia; age + BMI + smoking habit). (2) Multivariate analysis including age, BMI, and time since second inoculation together with variables with significant association (*p* ≤ .05) at multivariate model 1, analysed one by one as regressors (age + BMI + time since second inoculation + waist circumference; age + BMI + time since second inoculation + hypertension; age + BMI + time since second inoculation + dyslipidaemia; age + BMI + time since second inoculation + smoking habit). (3) Multivariate analysis including all statistically significant variables of the multivariate model 1 with the addition of gender, age, and BMI, as regressors in one single model. (4) Multivariate analysis including all statistically significant variables of the multivariate model 1 with the addition of gender, age, BMI, and total body fat mass (quartiles) as regressors in one single model. The variables results were added in the table, reporting their B and 95% CI [*R* ^2^]. For the analysis, a *p*‐IN = 0.05 and a *p*‐OUT = 0.10 were used. The effect estimate is reported as the coefficient of determination *R* ^2^, which informs on how much the model explains the variance of the dependent variable. Variance inflation factor (VIF) values were lower than 4.0, suggesting the absence of multicollinearity between included variables.^10^ The results were considered statistically significant when *p* < 0.05.

## 3 RESULTS

### 3.1 Study population

Eighty‐six subjects were enrolled in the present study in January and February 2021 among healthcare workers adhering to the vaccination campaign of Policlinico Umberto I Hospital, Rome, Italy. The clinical characteristics of the participants are summarized in Table 1. Briefly, the age was 29 (17), 39.5% male, BMI 22.4 (5.5) kg/m^2^, all were Caucasian. Of total, 31.7% was a current smoker, 15.3% was hypertensive on pharmacological treatment (of which almost 100% on angiotensin‐converting enzyme inhibitors/angiotensin II receptor blockers, ACEI/ARBs), only 2.4% was diabetic and 7.1% dyslipideamic. Of total, 76.8% had undergone routine influenza virus vaccination in the preceding 12 months, as recommended per hospital policy. A small panel of routine biochemical tests was normal for all participants and is summarized in Table 1.

**TABLE 1:** Descriptive characteristics of study population

	Mean ± *SD* or median (IQR) or *N* (%)
Age (years)	29 (17)
Weight (kg)	66 (17.8)
Gender (female, %)	60.50%
BMI (kg/m^2^)	22.4 (5.5)
Waist circumference (cm)	91 ± 11.3
Hip circumference (cm)	99 ± 9
Waist‐to‐hip ratio	0.9 (0.09)
Fat mass (kg)	16.1 (7.6)
Lean mass (kg)	48.4 (16.3)
Time (days)	22 (5)
Smoking habit *N* (%)	26 (31.7%)
Hypertension *N* (%)	13 (15.3%)
Type 2 diabetes *N* (%)	2 (2.4%)
Dyslipidaemia *N* (%)	6 (7.1%)
Creatinine (mg/dl)	0.86 (0.25)
BUN (mg/dl)	30.6 ± 8.7
ALT(U/L)	15 (13.5)
AST(U/L)	21 (9)
Total cholesterol (mg/dl)	200 (55)
HDL cholesterol (mg/dl)	58 (26)
Triglycerides (mg/dl)	85 (71)
Uric acid (mg/dl)	4.5 (1.8)
CRP (mg/L)	700 (1100)

Abbreviations: ALT, alanine aminotransferase; AST, aspartate aminotransferase; BUN, blood urea nitrogen; CRP, C‐reactive protein; F, female; IQR, interquartile range; *N*, number.

Regarding adiposity measures, 63.1% had a normal weight, 27.4% overweight, and 9.5% obesity according to BMI cut‐offs. However, central obesity was observed in 60.9% (*n* = 53) of subjects out of 78 for whom waist circumference measurements had been recorded. The cutoff to determine central obesity was 80 cm for women, and 94 cm for men.^11^

### 3.2 Safety

All adverse events following the vaccine inoculation were recorded with the means of a structured interview, and 65.9% (*n* = 56) of participants complained of some adverse events following the first inoculation. Of these, 50 reported of pain or pruritus in the site of inoculation, 10 of headache, fatigue, or malaise, three of low‐grade fever, two of dyspnoea, and five of other minor adverse events. Following the second inoculation, 78.2% (*n* = 61) reported some adverse event, of which 44 reported of pain or pruritus in the site of inoculation, 28 of headache, fatigue, or malaise, 21 of low‐grade fever, and eight of other minor adverse events. No major adverse event requiring hospitalization was recorded at any time point. Adiposity parameters such as higher waist circumference, waist‐to‐hip ratio, BMI, or body fat were not associated with more adverse events (data not shown).

### 3.3 Efficacy

**FIGURE 1:**
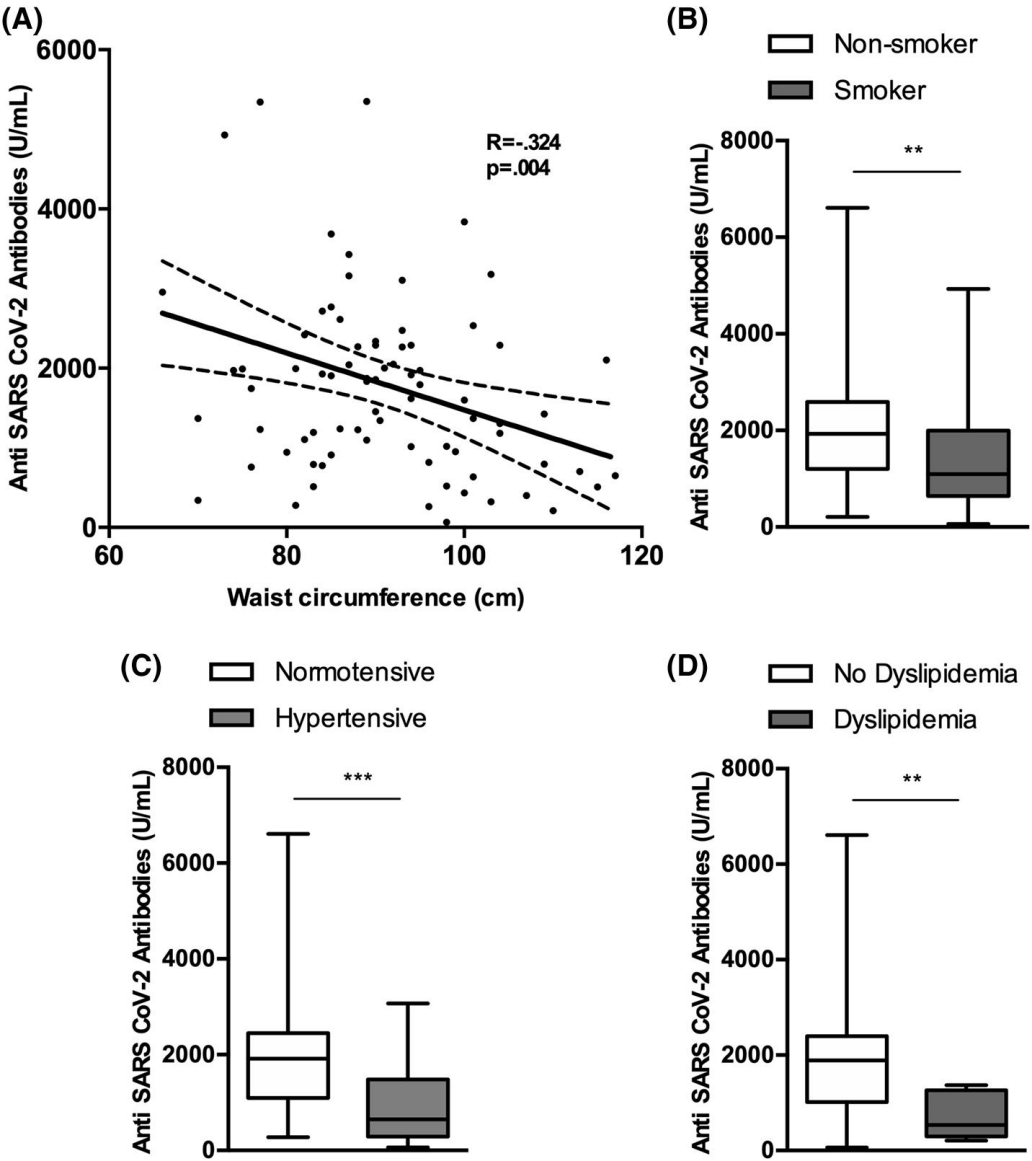
Serological response in relation to selected cardiometabolic conditions and risk factors. (A) Scattered dot‐plot representing the correlation between anti‐SARS‐CoV‐2 antibody titres and waist circumference. *p* is from a univariate linear regression analysis, with Ab titres as the dependent variable and waist circumference as the independent variable; (B) smokers; (C) hypertensive subjects; and (D) dyslipideamic subjects showed lower Ab titres compared to non‐smokers, normotensive, and those with a normal lipid profile, respectively. *p < 0.05, **p < 0.01, **p < 0.001, ***p < 0.000

Patients with higher waist circumference had significantly lower SARS‐CoV‐2 antibody titres (*R* = −0.324, *p* = 0.004; Figure 1A). Interestingly, obesity identified as a BMI ≥ 30 kg/m^2^ was not associated with a lower SARS‐CoV‐2 antibody titres (*p* = 0.524; data not shown). Furthermore, subjects with a smoking habit had lower Ab levels compared to those who were not current smokers [1099 (1350) versus 1921 (1375), respectively, *p* = 0.007; Figure 1B], and the same was for those who were hypertensive compared to those who were not [650 ± 1192 versus 1911 (1364), respectively, *p* = 0.001; Figure 1C], and those with dyslipidaemia compared to those who had a normal lipid profile and were not on lipid lowering drugs [534 (972) versus 1872 (1406), respectively, *p* = 0.005; Figure 1D].

Regression analysis showed that the presence of central obesity (as waist circumference measurement or waist‐to‐hip ratio) was associated with lower antibody concentration following the vaccine, as were hypertension, dyslipidaemia, and smoking habit (Table 2). Moreover, the time since the second vaccine inoculation at which the Ab titres were evaluated was significantly associated with a decline in serum SARS‐CoV‐2 Ab (Table 2). The presence of side effects following the first or second inoculation was not associated to different Ab titres, neither was flu vaccination in the preceding 12 months (Table 2).

**TABLE 2:** Univariate and multivariate linear regression analyses for anti‐SARS‐CoV‐2 antibody titre following vaccination

Variables	Univariate	Multivariate			
		1	2	3	4
	B (95% CI) *p*‐value (*R* ^2^)	B (95% CI) *p*‐value (*R* ^2^)	B (95% CI) *p*‐value (*R* ^2^)	B (95% CI) *p*‐value (*R* ^2^ = 0.361)	B (95% CI) *p*‐value (*R* ^2^ = 0.365)
Gender (female)	12.27 (−528.82 to 553.37) *p* = 0.964 (0.000024)			−22.854 (−660.9 to 615.223) *p* = 0.94	44.427 (−659.52 to 748.374) *p* = 0.9
Age (years)	−19.84 (40.55 to −0.865) *p* = 0.060 (0.041)			32.326 (−1.929 to 66.58) *p* = 0.06	34.688 (−3.001 to 72.377) *p* = 0.071
**Anthropometric parameters**					
Weight (kg)	−5.21 (−23.38 to 12.95) *p* = 0.570 (0.004)				
BMI (kg/m^2^)	−36.80 (−101.26 to 27.65) *p* = 0.259 (0.015)			53.858 (−47.85 to 155.562) *p* = 0.29	69.606 (−44.099 to 183.311) *p* = 0.226
Waist circumference (cm)	−**35.75** (**−59.58 to −11.94**) ***p* = 0.004** (**0.105**)	**−43.53 (−81.71 to −5.35) *p* = 0.026 (0.117)**	**−38.03 (−75.22 to −0.843) *p* = 0.045 (0.186)**	**−42.429 (−80.37 to −4.493) *p* = 0.03**	**−41.17 (−83.228 to 0.889) *p* = 0.055**
Hip circumference (cm)	−31.04 (−62.76 to 0.687) *p* = 0.055 (0.048)				
Waist‐to‐hip ratio	−**3336.95** (**−6466.91 to −206.99**) ***p* = 0.037** (**0.056**)	−2323.68 (−5836.17 to 1188.79) *p* = 0.192 (0.078)			
**Vaccine data**					
Time for Abs evaluation (days)	−**85.56** (**−139.26 to −31.87**) ***p* = 0.002** (**0.108**)	**−75.91 (−134.07 to −17.90) *p* = 0.011 (0.120)**		−**90.51 (153.72 to −27.31) *p* = 0.006**	**−89.178 (−155.49 to −22.863) *p* = 0.009**
Previous flu vaccine	−349.00 (−996.73 to 296.93) *p* = 0.285 (0.014)				
Side effects at the first inoculation	−412.38 (−969.78 to 145.01) *p* = 0.145 (0.025)				
Pain at the site of the first inoculation	444.35 (−83.18 to 917.89) *p* = 0.098 (0.032)				
Headache after the first inoculation	364.74 (456.76–1186.25) *p* = 0.380 (0.009)				
Fever after the first inoculation	10.63 (1431.23–1452.50) *p* = 0.988 (0.000003)				
Side effects at the second inoculation	116.53 (−547.01 to 780.07) *p* = 0.727 (0.002)				
Pain at the site of the second inoculation	470.48 (−48.84 to 989.81) *p* = 0.075 (0.037)				
Headache after the second inoculation	−43.84 (−608.35 to 520.66) *p* = 0.878 (0.000284)				
Fever after the second inoculation	−26.99 (−642.79 to 588.80) *p* = 0.931 (0.00009)				
**Medical history**					
Type 2 diabetes	−144.98 (−1919.72 to 1620.76) *p* = 0.876 (0.000321)				
Hypertension	−**1033.16 (−1741.85 to −324.46**) ***p* = 0.005** (**0.092**)	−**973.27** (**−1882.55 to −63.99**) ***p* = 0.036** (**0.096**)	−**1131.84** (**−2005.81 to −257.87**) ***p* = 0.012** (**0.188**)	−1113.4 (−2046 to 181.17) *p* = 0.02	**−1142.11** (**−2139.9 to −144.31**) ***p* = 0.026**
Dyslipidaemia	−**1223.43** (**−2233.83 to −213.02**) ***p* = 0.018** (**0.065**)	−**1057.45** (**−2109.55 to −5.36**) ***p* = 0.049** (**0.090**)	−**1022.84** (**−2039.56 to −6.11**) ***p* = 0.049** (**0.162**)	−645.31 (**−**1675 to 384.481) *p* = 0.22	−655.898 (−1709.7 to 397.864) *p* = 0.218
Smoking habit	−**680.93** (**−1249.06 to 112.80**) ***p* = 0.019** (**0.066**)	−**659.24** (**−1236.27 to −82.21**) ***p* = 0.026** (**0.096**)	**−808.67** (**−1366.80 to −250.54**) ***p* = 0.005** (**0.192**)	**−698.28** (**−1228.87 to 167.69**) ***p* = 0.011**	**−698.28** (**−1228.87 to −167.69**) ***p* = 0.011**
**Biochemical data**					
BUN (mg/dl)	−13.99 (−43.05 to 15.05) *p* = 0.341 (0.011)				
Creatinine (mg/dl)	−11.95 (−1315.65 to 1291.75) *p* = 0.985 (0.000004)				
AST U/L	−22.06 (−51.80 to 7.68) *p* = 0.144 (0.025)				
ALT U/L	−11.27 (−32.1 to 9.66) *p* = 0.287 (0.013)				
Total cholesterol (mg/dl)	−1.53 (−7.41‐4.34) *p* = 0.604 (0.003)				
HDL (mg/dl)	2.53 (10.81–15.87) *p* = 0.70 (0.002)				
Triglycerides (mg/dl)	−2.11 (−5.05 to 0.823) *p* = 0.156 (0.024)				
Uric acid (mg/dl)	−52.86 (−248.45 to 142.73) *p* = 0.592 (0.03)				
CRP mg/L	−0.045 (−0.223 to 0.133) *p* = 0.616 (0.003)				
**Body composition parameters**					
Total body fat mass (quartile)	−242.55 (−486.87 to 1.76) *p* = 0.052 (0.047)				−99.678 (−474.32 to 274.959) *p* = 0.597
Total body lean mass (quartile)	−34.15 (284.25 to 215.94) *p* = 0.786 (0.001)				

*Note:* To build a multivariate linear regression model with antibody titre as the dependent variable, we used a enter method approach (all the independent variables included in the same regression equation) and investigated the following variables/models: (1) multivariate analysis including age and BMI together with variables with significant univariate association (*p* value ≤ 0.05) analysed one by one as regressors (age + BMI + waist circumference; age + BMI + waist to hip ratio; age + BMI + time for Abs evaluation; age + BMI + hypertension; age + BMI + dyslipidaemia; age + BMI + smoking habit). (2) Multivariate analysis including age, BMI, and time for Abs evaluation together with variables with significant association (*p* value ≤ 0.05) at multivariate model 1, analysed one by one as regressors (age + BMI + time for Abs evaluation + waist circumference; age + BMI + time for Abs evaluation + hypertension; age + BMI + time for Abs evaluation + dyslipidaemia; age + BMI + time for Abs evaluation + smoking habit). (3) Multivariate analysis including all statistically significant variables of the multivariate model 1 with the addition of gender, age and BMI, as regressors in one single model. (4) Multivariate analysis including all statistically significant variables of the multivariate model 1 with the addition of gender, age, BMI, and total body fat mass (quartiles) as regressors in one single model. The variables results were added in the table, reporting their OR and 95% CI [*R* ^2^]. For the analysis, a *p*‐IN = 0.05 and a *p*‐OUT = 0.10 were used. The effect estimate is reported as the coefficient of determination *R* ^2^, which informs on how much the model explains the variance of the dependent variable. The results were considered statistically significant when *p* < 0.05. Pearson coefficient values are highlighted in bold when correlation was statistically significant at the *p* < 0.05 level and below.

Abbreviations: ALT, alanine aminotransferase; AST, aspartate aminotransferase; BMI, body mass index; BUN, blood urea nitrogen; CI, confidence interval; CRP, C‐reactive protein.

Each parameter showing a significant univariate association with Ab titres were included in a multivariate analysis together with age and BMI, factors possibly affecting the association, analysed one by one as regressors (Table 2, multivariate model 1). Waist circumference, time elapsed since vaccination, hypertension, diabetes, and dyslipidaemia retained a significant association. As the time factor was strongly associated with the Ab titres, we conducted a second multivariate analysis including each parameter with a significant association at multivariate model 1, together with BMI, age and time as factors likely playing a relevant role in the association, analysed one by one as regressors (Table 2, multivariate model 2). The addition of time in the model did not influence the results. A third multivariate model including all the statistically significant variables of the multivariate model 1 together, controlling for gender, age, and BMI, showed that waist circumference, time since vaccination, hypertension, and smoking habit were still significantly associated with Ab titres (Table 2, multivariate model 3). To test whether body fat underlay the significant association between waist circumference and the lower Ab levels in response to vaccination, a fourth multivariate model included DXA measured body fat on top of all parameters included in Model 3, showing that the association of waist circumference was lost. Conversely, the association with hypertension and smoking habit were maintained (Table 2, multivariate model 4).

## 4 DISCUSSION

Herein, we report that central obesity, independent of BMI is associated with lower Ab titres following a COVID‐19 mRNA vaccine. This could be due to a number of reasons, one of which is the metabolic derangements that often come with visceral adiposity, together with the immune dysfunction that has been reported in patients with obesity.^12^, ^13^ In fact, strong evidence supports the fact that obesity is also associated with poor seroconversion upon some vaccine administration,^14^ together with increased risk of infection even when the seroconversion seems robust.^15^ A recent study has shown that higher BMI is associated with lower Ab titres in response to COVID‐19 vaccine in Italian healthcare workers.^16^ Although pointing in the same direction, our findings slightly differ from this study, as we could not find any significant association between BMI and SARS‐CoV‐2 Ab titres following vaccination. This could be due to a narrower distribution in terms of BMI in our cohort, but it should also be highlighted that the kits to detect Ab titres were different, as was the timing of the Ab measurement (1 week). It is worth noting that Ab titres tend to rapidly decline following the vaccine with a kinetics not entirely elucidated yet and potentially different in some subpopulations.^17^ Moreover, it is acknowledged that BMI poorly describes actual body fat excess, which is the definition of obesity according to the World Health Organization,^7^ and a simple parameter such as waist circumference is shown to be even more associated to chronic low‐grade inflammation and cardiovascular disease, morbidity, and mortality as opposed to BMI.^18^ Indeed, we have recently shown that visceral fat is the strongest predictor of the need of intubation following COVID‐19 infection,^1^ suggesting that the same trend might apply to the vaccine response, where an accumulation in central fat may impact Ab levels, possibly hindering the response of these patients against an eventual subsequent infection. It is, therefore, of utmost importance to determine in future studies whether cell mediated immunity is maintained even when Ab titres decline in this population. It is worth noting that that when including DXA measured body fat as covariate in a multivariate analysis, the significant association between waist circumference and lower Ab titres was lost, suggesting that body fat may underlie the link. However, given that the effect size was only marginally improved by this addition, a definitive conclusion cannot be drawn.

We also report that smoking and hypertension were strongly associated with lower Ab titres. Interestingly, it was previously reported that Ab titres following influenza vaccination decline more rapidly in smokers, through an unknown mechanism.^19^ More generally, the habit of smoking is associated with a dysfunctional immune system, linked with both autoimmune disease and reduced response to infections.^20^ Similarly, hypertension and an inappropriate response to vaccinations might have a common root into a dysfunctional immune system according to recent evidence.^21^ Moreover, similar to central obesity, the presence of hypertension was previously found to be related to worse COVID‐19 outcomes,^22^, ^23^ suggesting that the same cardiometabolic features linked to higher morbidity and mortality upon COVID‐19 infection may be involved in the development of an immunological response to the vaccination against it. To date, no association between smoking or hypertension and reduced protection following COVID‐19 vaccine has been reported.

It has been recently reported that elderly patients and those with cancer also develop lower Ab titres following the same mRNA COVID‐19 vaccine.^24^, ^25^ These findings suggest that some, more vulnerable, subpopulations may respond in a different way to this vaccine. Whether this is linked to its nucleic acid nature or to the virus itself is yet to be elucidated, and studies comparing different types of vaccines are necessary.

Our study has several limitations. First, the time of evaluation ranged between 1 and 4 weeks following the second vaccine inoculation, introducing time as a possible bias. However, when the evaluations were controlled for this factor, they still yielded the same results. Second, the sample size of our study population was relatively small, and patients with multiple comorbidities were underrepresented, possibly hindering some of the results. The primary aim of the present study was to investigate the link between the serological response following COVID‐19 vaccination and adiposity parameters, with the assessment of a possible link with other cardiometabolic conditions and risk factors as a secondary aim. Therefore, the finding that smoking and hypertension and/or treatment with ACEi/ARBs are associated with lower Ab titres warrants further, adequately powered, confirmatory studies. Further, the BMI range distribution was relatively narrow, although reflecting the prevalence of overweight and obesity in the general Italian population.^26^ This could have hampered possible significant results regarding BMI differences. However, we collected other relevant adiposity parameters, such as DXA‐derived body fat and waist and hip circumference, which showed a wide distribution within our study population. Due to the relatively young age and healthy lifestyle of our cohort of healthcare workers, DXA diagnosed sarcopenia was not a feature adequately represented to allow for investigation. It is however well established that sarcopenia represents a significant risk for higher morbidity and mortality,^27^ and its link with the serological response to COVID‐19 vaccination should be elucidated. Finally, we investigated the Ab titres shortly after the second inoculation, and following the same patients over time is warranted in order to investigate the Ab kinetics according to body composition and other possibly relevant factors. Moreover, cell‐mediated immunogenicity warrants further attention, and studies including these outcomes are therefore needed. Certainly, anti‐SARS‐CoV‐2 Ab titres following vaccination cannot predict the likelihood of developing COVID‐19 at this point, and low but measurable levels may as well be highly protective against infection. However, neutralizing Ab titres correlate with protection against several viruses including SARS‐CoV‐2,^28^ ^,^ ^29^ and the finding that central obesity, hypertension, and smoking are associated with lower Ab concentration shortly after the vaccination warrants further attention, as this may mean that these subjects respond in a different way to the same vaccination and may require different vaccine booster schedules over time.

Our study also features some strengths. This is, to the best of our knowledge, the first study reporting data on the immunogenicity of a COVID‐19 vaccine according to central obesity indices. Healthcare professionals were the first being vaccinated across all countries, so these are the earliest real‐life findings becoming available. Waist circumference as a marker of central obesity does not require additional instrumental tests, it is cheap and easy to collect, and it therefore possesses a possible immediate clinical applicability. Clinical history was acquired with the means of a standardized structured interview allowing for a thorough and complete collection, and the adverse events were reported 3 days after the two vaccine inoculations, limiting the risk of recall bias.

With the general population now being vaccinated, more and more subjects with central and general obesity will receive the vaccine, and very soon booster schedules will need to be planned. The fact that the Ab response is lower in certain subjects shortly after the second inoculation must lead to a highly vigilant approach, as medium and long‐term data will become available only when the schedule will have been necessarily set already.

## CONFLICT OF INTERESTS

The authors declare that there are no conflict of interests.

## ETHICS STATEMENT

The study was approved by the local IRB (prot. CE 6228), conducted in accordance with the Declaration of Helsinki and the Good Clinical Practice. Written informed consent was obtained from all study participants before enrolment.

## AUTHOR CONTRIBUTIONS

Mikiko Watanabe, Angela Balena, and Dario Tuccinardi collaborated equally on this work and are joint first authors. Mikiko Watanabe, Angela Balena, Dario Tuccinardi, Silvia Manfrini, Stefania Mariani, Carla Lubrano, Andrea Lenzi, Claudio Mastroianni, Lucio Gnessi contributed to the conception and design of the work. Mikiko Watanabe coordinated the work, supported by Silvia Manfrini, Stefania Mariani, Carla Lubrano, Andrea Lenzi, Claudio Mastroianni, Lucio Gnessi. Angela Balena, Rossella Tozzi, Renata Risi, Davide Masi, Alessandra Caputi, Rebecca Rossetti, Maria Elena Spoltore, Valeria Filippi, Elena Gangitano acquired the data. Dario Tuccinardi, Renata Risi, and Carla Lubrano conducted the statistical analysis. All authors provided substantial scientific input in interpreting the results, drafting and or reviewing the manuscript. Mikiko Watanabe is the guarantor. The corresponding author attests that all listed authors meet authorship criteria and that no others meeting the criteria have been omitted.

### PEER REVIEW

The peer review history for this article is available at https://publons.com/publon/10.1002/dmrr.3465.

## Data Availability

Due to concerns for participant privacy, data are available only upon reasonable request to the corresponding author.
